# Demographics of Patients Traveling Notable Distances to Receive Total Knee Arthroplasty

**DOI:** 10.5435/JAAOSGlobal-D-22-00159

**Published:** 2022-08-12

**Authors:** Matthew Orringer, Heather Roberts, Derek Ward

**Affiliations:** From the School of Medicine (Dr. Orringer), and the Department of Orthopaedic Surgery (Dr. Roberts, Dr. Ward), University of California, San Francisco, Francisco, CA.

## Abstract

Although disparities exist in patient access to and outcomes after total knee arthroplasty (TKA), there are limited data regarding the relationship between travel distance and patient demographics or postoperative complications.

**Methods:**

We identified patients who underwent TKA in Florida and New York between 2006 and 2014 using the Healthcare Cost and Utilization Project State Inpatient Databases. The American Hospital Association and UnitedStatesZipCodes.org Enterprise databases were further used to calculate distance traveled from patient population-weighted zip code centroid points to the hospitals at which they underwent TKA. Patients were grouped by travel distance: 25 to 50, 50 to 100, 100 to 500 miles, and greater than 500 miles. Patient demographic characteristics and postoperative outcomes were compared between the travel distance groups.

**Results:**

Older age, increased medical comorbidities, White race, Medicare insurance coverage, and living in zip codes with greater mean income levels were associated with greater travel distance (*P* < 0.001). There were no clinically significant relationships between various postoperative complications and distance traveled.

**Discussion:**

Certain demographic variables are associated with increased travel distance to undergo TKA. These relationships were most pronounced at the extremes of distance traveled (>500 miles). These differences may indicate that specific patient groups are either electing to or being forced to travel notable distances for orthopaedic care. Additional research is needed to determine the causative mechanisms underlying these findings.

Total knee arthroplasty (TKA) is a frequently used procedure in the treatment of osteoarthritis.^1^ There are robust data describing disparities in both patient access to and outcomes after joint arthroplasty surgery. One review found that White patients with greater socioeconomic status had more favorable postoperative results and were more likely to undergo total joint arthroplasty surgery, whereas Black and Hispanic patients had worse outcomes and were less likely to receive total joint arthroplasty (TJA).^2^ Similarly, Ihekweazu et al^3^ found that patients who were Black, Hispanic, female, and older or had later procedure end times had longer length of stay after TKA while factors such as being married and undergoing TKA during a holiday week were associated with reduced length of stay. Additional studies have produced similar results for race and financial resources.^4-6^

However, there exists limited research investigating the association between travel distance and patient demographics and postoperative outcomes. One study from a tertiary care center found that patients who traveled farther for TKA were more likely to be male, be younger, and have disease processes related to inflammatory arthritis or neoplasms.^7^ Despite these differences, patients had similar postoperative outcomes, including revision surgery and revision rates, across the varying travel distance groups in the study.

The purpose of our study was to investigate the relationship between distance traveled to undergo TKA and various patient demographic characteristics. We additionally evaluated how adverse postoperative outcomes relate to patient travel distance. We hypothesized that patient factors such as patient age, income level, race, insurance provider, and medical comorbidities would be associated with distance traveled for TKA. Specifically, we hypothesized that patients with additional medical comorbidities, those with commercial insurance coverage, and White patients would have increased travel distance. We similarly hypothesized that patients traveling farther to undergo TKA would be more likely to have postoperative adverse outcomes because we expected that this group of patients may have additional preexisting comorbid medical conditions.

## Methods

We identified patients who underwent TKA from 2006 to 2014 in Florida and New York using the Healthcare Cost and Utilization Project (HCUP) State Inpatient Databases. Patients who were younger than 18 years were excluded. In addition, those patients who were admitted through the emergency department, those who underwent bilateral TKA, and those for whom there were incomplete zip codes were excluded from analysis.

To gather the geographic coordinates of the hospitals at which patients underwent TKA, we used the American Hospital Association database. We linked this database with HCUP based on entries with the same hospital ID number. The HCUP database provided patient demographic data including age, race, sex, resident zip code, insurance status, and medical comorbidities (Charlson Comorbidity Index, see Appendix A, http://links.lww.com/JG9/A229).^8^ Furthermore, HCUP included patients’ postoperative complication data (see Appendix B, http://links.lww.com/JG9/A229).

We used the UnitedStatesZipCodes.org Enterprise data set to determine population-weighted zip code centroid points for each patient.^9^ Next, we calculated the distance between patient population-weighted zip code centroid points to the hospital at which they received care. This was done using the Stata “geodist” command, which uses the great circle equation and is accurate within 0.5%. This equation calculates the shortest distance between two points along a spherical surface.^10^

We focused our analysis on patients who traveled at least 25 miles to receive care to best assess differences in access to care and thus excluded patients who traveled less than 25 miles. We then divided patients into four groups based on travel distances: 25 to 50, 50 to 100, 100 to 500 miles, and greater than 500 miles traveled.

We first analyzed the relationship between distance traveled and patient age and Charlson Comorbidity Index using one-way analysis of variance tests. We then assessed the relationship between travel distance and categorical demographic variables of race, insurance provider, and zip code median income quartile using chi square tests. Finally, we used travel distance as a continuous variable to assess differences in postoperative complication rates as a function of distance traveled. These analyses were conducted using logistic regression. All analyses were conducted using Stata MP 16 analytical software (StatCorp, LLC), and statistical significance was set at *P* < 0.05.

## Results

There were 44,062 patients included in our analysis. These patients were of an average age of 66.44 years, with a mean Charlson Comorbidity Index (CCI) score of 2.89. Women represented 58.4% of the patients, and 69.2% of the patients were from Florida. White patients comprised 88.5% of the patients, with Black (4.4%) and Hispanic (3.2%) patients as the next most represented. Medicare insurance coverage was the most common (60.6%) in our cohort of patients while 31.2% of the patients had commercial insurance plans. Patients were distributed across zip code income quartiles as follows: 33.0% in the lowest quartile, 33.0% in the next lowest quartile, 18.8% in the second highest group, and 14.1% in the top income quartile. Table 1 summarizes these data.

**Table 1:** Demographic Data

Age: mean ± SD	66.44 (10.10)
CCI: mean ± SD	2.89 (1.51)
Sex: n (%)	
Male	18,317 (41.6)
Female	25,745 (58.4)
Race: n (%)	
Asian	144 (0.3)
Black	1948 (4.4)
Hispanic	1415 (3.2)
Native American	82 (0.2)
White	38,993 (88.5)
Missing or other	1480 (3.4)
Insurance provider: n (%)	
Commercial	13,753 (31.2)
Medicaid	1005 (2.3)
Medicare	26,709 (60.6)
Other	2595 (5.9)
Hospital state: n (%)	
Florida	30,495 (69.2)
New York	13,567 (30.8)
Zip code income quartile: n (%)	
1 (lowest)	14,521 (33.0)
2	14,545 (33.0)
3	8287 (18.8)
4 (highest)	6190 (14.1)
Null	519 (1.2)

A statistically significant relationship was observed between patient age and travel distance (*P* < 0.0001). The average patient age increased in a step-wise manner as patient travel distance increased. This trend was particularly pronounced in the greater-than-500-miles-traveled group, in which patients were of an average age of 71.0 years compared with 67.5 years in the 100 to 500 miles group. Similarly, CCI was significantly associated with travel distance (*P* < 0.0001). Mean CCI was lowest in the 25 to 50 miles group (2.85) and highest in the greater-than-500-miles group (3.2). These data are summarized in Table 2.

**Table 2:** Travel Distance Versus Age and CCI

Distance Group	Frequency	Mean Age (SD)	Mean CCI (SD)
25-50 miles	29,938	66.0 (10.0)	2.85 (1.5)
50-100 miles	10,053	67.1 (10.1)	2.95 (1.5)
100-500 miles	3148	67.5 (10.6)	2.95 (1.5)
>500 miles	923	71.0 (8.6)	3.2 (1.3)
		*P* < 0.0001	*P* < 0.0001

Patient travel distance and insurance provider were significantly related (*P* < 0.001). Similar to the age, CCI, and race data, the relationship was most pronounced among patients in the greater-than-500-miles group. The relative proportions of patients who traveled between 25 and 500 miles to receive care were similar to the overall composition of the cohort. However, among patients who traveled at least 500 miles for TKA, 79.3% of the patients had Medicare coverage (versus 60.6% overall) and 18.1% had commercial insurance plans (versus 31.2% overall). In addition, zero of the 1005 patients covered by Medicaid traveled 500 miles for TKA. Table 3 includes these data.

**Table 3:** Travel Distance Versus Insurance Provider (Percent)

Distance Group	Commercial	Medicaid	Medicare	Other
25-50 miles	32.9	2.5	58.7	6.0
50-100 miles	27.4	1.9	64.3	6.4
100-500 miles	31.3	2.3	62.1	4.3
>500 miles	18.1	0	79.3	2.6
Total	31.2	2.3	60.6	5.9

*P* < 0.001.

We found a statistically significant interaction between travel distance and patient race (*P* < 0.001). White patients comprised 91.2% of the longest travel distance group compared with 88.5% of the overall cohort of patients. Conversely, Black patients made up only 2.2% of the greatest travel distance group versus 4.4% of the total sample of patients. Table 4 summarizes these data.

**Table 4:** Travel Distance Versus Race (Percent)

Distance Group	Asian	Black	Hispanic	Other	Native American	White
25-50 miles	0.3	4.6	3.4	3.1	0.2	88.4
50-100 miles	0.3	3.9	2.3	4.0	0.2	89.5
100-500 miles	0.6	5.4	4.5	4.0	0.2	85.4
>500 miles	0.2	2.2	2.7	3.6	0.1	91.2
Total	0.3	4.4	3.2	3.4	0.2	88.5

*P* < 0.001.

Patient travel distance was significantly associated with patient zip code median income quartile (*P* < 0.001). Patients in the lowest two income groups were underrepresented in both the 100 to 500 miles and greater-than-500-miles groups. Notably, only 9.4% of the patients in the lowest income group were in the top travel distance group despite making up 33.0% of the overall cohort of patients. Conversely, patients from higher earning zip codes were more likely to travel greater distances for care. Patients from the third highest income group made up 28.7% of the highest travel distance group (versus 18.8% of the total cohort). Patients from the top earning zip codes comprised the largest proportion of patients traveling both 100 to 500 miles (32.0%) and greater than 500 miles (34.5%) despite being the smallest patient cohort overall (14.1%). Table 5 summarizes these data.

**Table 5:** Travel Distance Versus Zip Code Income (Percent)

Distance Group	1 (Lowest)	2	3	4 (Highest)	Null
25-50 miles	33.1	33.1	19.5	13.1	1.2
50-100 miles	38.4	35.9	14.9	9.3	1.4
100-500 miles	20.8	24.2	22.2	32.0	0.9
>500 miles	9.4	26.4	28.7	34.5	1.0
Total	33.0	33.0	18.8	14.1	1.2

*P* < 0.001.

Finally, we analyzed the relationship between patient travel distance and postoperative complications. We evaluated 13 outcomes and found that only two were significantly associated with travel distance: mechanical malfunction within 365 days (*P* = 0.001) and readmission within 30 days (0.047). However, although statistically significant, the magnitude of the effect found for each of these variables was small. These data are presented in Table 6.

**Table 6:** Travel Distance Versus Postoperative Adverse Outcomes

Outcome	Coefficient	*P*
Stroke	−0.0007	0.269
Death within 30 days	−0.0002	0.822
Death within 90 days	−0.0002	0.816
Death within 365 days	−0.0003	0.573
Cardiac complication within 30 days	0.0002	0.545
DVT within 60 days	0.0000	0.974
Genitourinary complication within 30 days	0.0001	0.772
Hematoma within 30 days	−0.0003	0.521
Mechanical malfunction within 365 days	−0.0007	0.001
Pulmonary embolism within 60 days	−0.0006	0.254
Prosthetic joint infection within 365 days	−0.0008	0.094
Respiratory complication within 30 days	0.0000	0.987
Readmission within 30 days	0.0003	0.047

DVT = deep vein thrombosis

## Discussion

Among the cohort of 44,062 patients, we found that travel distance to undergo TKA is associated with age, CCI, race, insurance provider, and zip code median income level. Although we found statistically significant relationships between distance traveled and rates of mechanical malfunction within 365 days of surgery and readmission within 30 days, these results are likely not clinically significant.

We found that patient age and CCI score were positively related to travel distance (*P* < 0.0001). Patients who traveled at least 500 miles were 71.0 years old on average as compared with the 25 to 500 miles groups, in which patient age ranged from 66.0 to 67.5 years. We also found that the mean CCI score increased from the shortest to longest travel distance groups with the 25 to 50 mile group having an average score of 2.85 versus the greater-than-500-miles group having an average score of 3.20. It is possible that older patients may be more likely to be retired and thus able to devote the extra time needed to obtain medical care at a specific, distant facility. In addition, it could be the case that older patients and those with increased medical comorbidities either elect to or are forced to undergo TKA at certain hospitals in which more specialized perioperative care is available. This supports our hypothesis that patients with greater medical comorbidities would travel farther for TKA. These data notably differ from prior research showing that younger patients traveled farther for TKA at a tertiary care center.^7^ Future research may involve identifying patients traveling great distances for TKA and interviewing these individuals to better understand the driving forces behind their decisions to receive care at a given hospital.

We found a statistically significant relationship between patient race and travel distance (*P* < 0.001). Black and Hispanic patients were relatively underrepresented in the farthest travel distance group while White patients were relatively overrepresented. These data may be the result of a number of factors. White patients may travel farther to receive TKA because they are more likely to live in rural areas with lower hospital density.^11^ Alternatively, these results may be related to socioeconomic status because White individuals have higher median income levels than Black or Hispanic individuals.^12^ Additional investigation is warranted to better understand the role that hospital density plays in determining patient travel distance as stratified by race.

We found that patients with Medicare insurance coverage traveled the farthest to receive TKA (*P* < 0.001). This is in contrast with past research and our hypothesis that patients with commercial insurance would travel greater distances to receive care. It could be the case that these results are driven by patient age. Medicare eligibility begins at the age of 65 years, and we found that older patients were more likely to travel notable distances for TKA. Notably, of the 1005 patients in our cohort who had Medicaid insurance, none traveled at least 500 miles for TKA. This suggests that financial resources likely play a role in determining which patients are able to travel great distances for surgery.

Patients residing in zip codes with higher median zip code income levels traveled farther to receive care than those living in areas with lower median income values (*P* < 0.001). Patients living in zip codes with the lowest median income level were underrepresented in both the 100 to 500 miles group and the greater-than-500-miles group. Conversely, those patients living in the wealthiest zip codes comprised 32.0% of the 100 to 500 miles traveled group and 34.5% of the greater-than-500-miles-traveled group despite making up only 14.1% of the overall cohort. This suggests that wealthier patients may be more able to spend the time and money necessary to travel to distant hospitals for care. These data indicate that patients may be at least in part seeking out specific care facilities as opposed to being forced to travel great distances for TKA. Thus, this may represent a patient-driven disparity in medical resource utilization.

We found statistically significant relationships between patient travel distance and mechanical malfunction within 365 days (*P* = 0.001) as well as readmission within 30 days of TKA (*P* = 0.047). However, the coefficient of effect was small for each of these associations (−0.0007 and 0.0003, respectively). Thus, these statistically significant results may be because of the large sample size of our study and likely do not indicate clinically significant differences. This refutes our hypothesis that patients who traveled farther to receive care would be more likely to experience postoperative complications. This may be, in part, due to the relative infrequency of postoperative complications after these procedures. These results are perhaps surprising given that we observed that patients with increased medical comorbidities traveled farther to receive care. Thus, it may be the case that patients are effectively choosing hospitals that meet their perioperative needs. These data support prior findings that travel distance did not affect patient outcomes after orthopaedic procedures.^3,13^

This study has several limitations. First, using large databases does not allow for analysis of patient-specific factors. Specifically, we were not able to analyze the reasoning that led patients to undergo TKA at a specific hospital. Although we are able to make inferences based on the results of this study, additional research is necessary to understand the causative mechanisms producing these data. Similarly, although population-weighted zip code centroid points have greater accuracy than simple zip code centroid coordinates, these data points may be less accurate in less populated zip codes and those that have a larger geographic area.^14^

In addition, we are not able to determine the role that urbanicity plays in our results because those patients residing in rural areas may be forced to travel farther for surgical treatment because of a lack of hospital density in their communities. Furthermore, the databases used in this study are not able to identify those patients who may need TKA but are unable to undergo the operation because of financial limitations and/or an inability to travel from their remote residence to a distant hospital center. Future research is needed to determine how to identify these patients to provide solutions to better facilitate surgical treatment.

In addition, the demographic makeup of our study included a disproportionately large number of White patients.^15,16^ This may be the result of broader disparities in the utilization of orthopaedic care.^2^ However, the relatively skewed racial composition of our study may limit the generalizability of the results.

## Conclusions

We found that older age, increased CCI score, White race, Medicare insurance coverage, and residence in a zip code with a higher median income level were associated with greater travel distance to undergo TKA. These relationships were especially pronounced at the extremes of travel distance (greater than 500 miles). Future research is needed to better understand the interplay between these variables and the patient-specific factors that lead individuals to select certain distant hospitals for TKA.
